# Highly selective fluorescent probe for detecting mercury ions in water†

**DOI:** 10.1039/d3ra02791k

**Published:** 2023-06-23

**Authors:** Yanfeng Shi, Bingxu Li, Zhifeng Wang, Yanhao Zhang, Zhibin Zhang, Xu Zhang, Fulin Li

**Affiliations:** a School of Municipal and Environmental Engineering, Shandong Jianzhu University Jinan China; b School of Architecture and Urban Planning, Shandong Jianzhu University Jinan China 15668303582@163.com +86 156 6830 3582; c Water Resources Research Institute of Shandong Province Jinan China fulinli@126.com

## Abstract

Mercury ion (Hg^2+^) is a well-known toxic heavy metal. It has become one of the most significant environmental pollutants in the world because of its serious physiological toxicity, persistence, easy migration, and high bioconcentration. Thus, the development of methods for monitoring Hg^2+^ is indispensable. Herein, we have designed and synthesized a new fluorescent probe, TPH, for the detection of Hg^2+^ in the water environment. The TPH probe could quantitatively detect Hg^2+^ between 0 and 5 μM (LOD = 16 nM), with a linear range of 0–2.5 μM. In addition, the TPH probe was used to monitor Hg^2+^ in water samples successfully. Thus, this probe is suitable for monitoring Hg^2+^ in the actual water environment.

## Abstract 2

A novel fluorescent TPH probe was developed to monitor Hg^2+^ in the water environment, which possessed many advantages, including good water solubility, excellent sensitivity (LOD = 16 nM), high selectivity, and excellent practical application.

## Graphical abstract

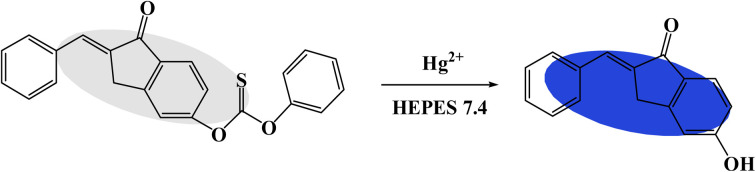

## 1 Introduction

Mercury has attracted attention from researchers owing to its strong toxicity and bioaccumulation.^1–4^ With the development of industry, especially gold mining, the burning of fossil and oil refining, mercury ion pollution is widely distributed in the environment.^5–7^ Through biological enrichment, mercury ions in water can be transformed into organic mercury ions with stronger toxicity and then enter the human body *via* the food chain.^8–10^ Even very small amounts of mercury ions have a severe impact on the human body, including the digestive system and kidneys, cognitive disorders, and even central nervous system damage.^11–16^ Consequently, the development of methods for the detection of mercury ions with simple synthesis and high selectivity and sensitivity is significant.

Recently, many methods have been reported for the detection of mercury ions (Hg^2+^), including gas chromatography, inductive coupled plasma mass spectroscopy, and atomic absorption spectrometry.^17–21^ However, most of the above-mentioned methods have some disadvantages, such as complex pretreatment, use of expensive instruments, and difficulty in realizing real-time and on-site monitoring.^22,23^ In contrast, the use of fluorescent probes has attracted significant attention due to their simple operation and high selectivity and sensitivity.^24–30^ Thus, an increasing number of fluorescent probes has been used to detect environmental heavy metal pollutants including Hg^2+^. However, the reported probes for monitoring Hg^2+^ still have some shortcomings, such as poor selectivity and water solubility and high detection limits (LODs).^31–35^ Therefore, new fluorescent probes need to be developed for monitoring Hg^2+^ in the environment with excellent selectivity and sensitivity and good water solubility.

Accordingly, herein, we synthesized the TPH probe, which was based on the TPC-OH dye as the fluorophore^36^ and phenyl thiochloroformate as the recognition receptor of Hg^2+^.^37,38^ Phenyl thiochloroformate possesses high selectivity for the detection of Hg^2+^, and thus the TPH probe could also achieve the specific and sensitive detection of Hg^2+^. The TPH probe exhibited the following excellent properties: (1) good water solubility, (2) excellent sensitivity (LOD = 16 nM), (3) high selectivity, and (4) excellent application in the environment. Thus, this probe will have a wide application prospect for monitoring Hg^2+^ in the environment.

## 2 Experimental

### 2.1 Materials and instruments

All chemical reagents were obtained from commercial sources and used without further purification. Absorption and fluorescence spectra were recorded on a UV-3101PC spectrophotometer and Horiba FluoroMax-4 spectrophotometer, respectively.

### 2.2 Synthesis of TPH probe

**Scheme 1:**
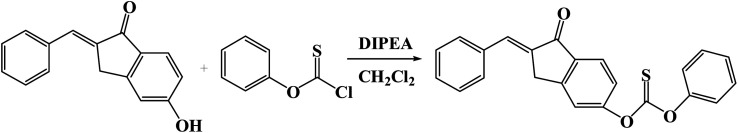
Synthesis of TPH probe.

TPC-OH dye (236 mg, 1 mmol) was dissolved in dry CH_2_Cl_2_ (15 mL), and then phenyl thiochloroformate (259 mg, 1.5 mmol) and *N*,*N*-diisopropylethylamine (DIPEA) (194 mg, 1.5 mmol) were added (Scheme 1). The mixed solution was stirred at 25 °C for 2 h.

The crude product was purified by column silica chromatography over silica gel using dichloromethane/petroleum ether (14 : 5) as the eluent to provide a faint-yellow pure solid product. ^1^H NMR (400 MHz, DMSO) *δ* (ppm): 7.94 (d, *J* = 5.6 Hz, 1H), 7.81 (d, *J* = 8.0 Hz, 2H), 7.68 (s, 1H), 7.58 (s, 1H), 7.55–5.52 (m, 4H), 7.48 (t, *J* = 8.0 Hz, 2H), 7.39 (t, *J* = 8.0 Hz, 3H), 4.21 (s, 2H). ^13^C NMR (100 MHz, DMSO) *δ* (ppm): 193.95, 192.55, 158.01, 153.62, 152.68, 136.23, 135.34, 135.21, 133.66, 131.32, 130.46, 130.42, 129.52, 127.57, 125.82, 122.74, 122.20, 120.54, 32.44.

## 3 Results and discussion

### 3.1 Spectral response of TPH probe

**Fig. 1:**
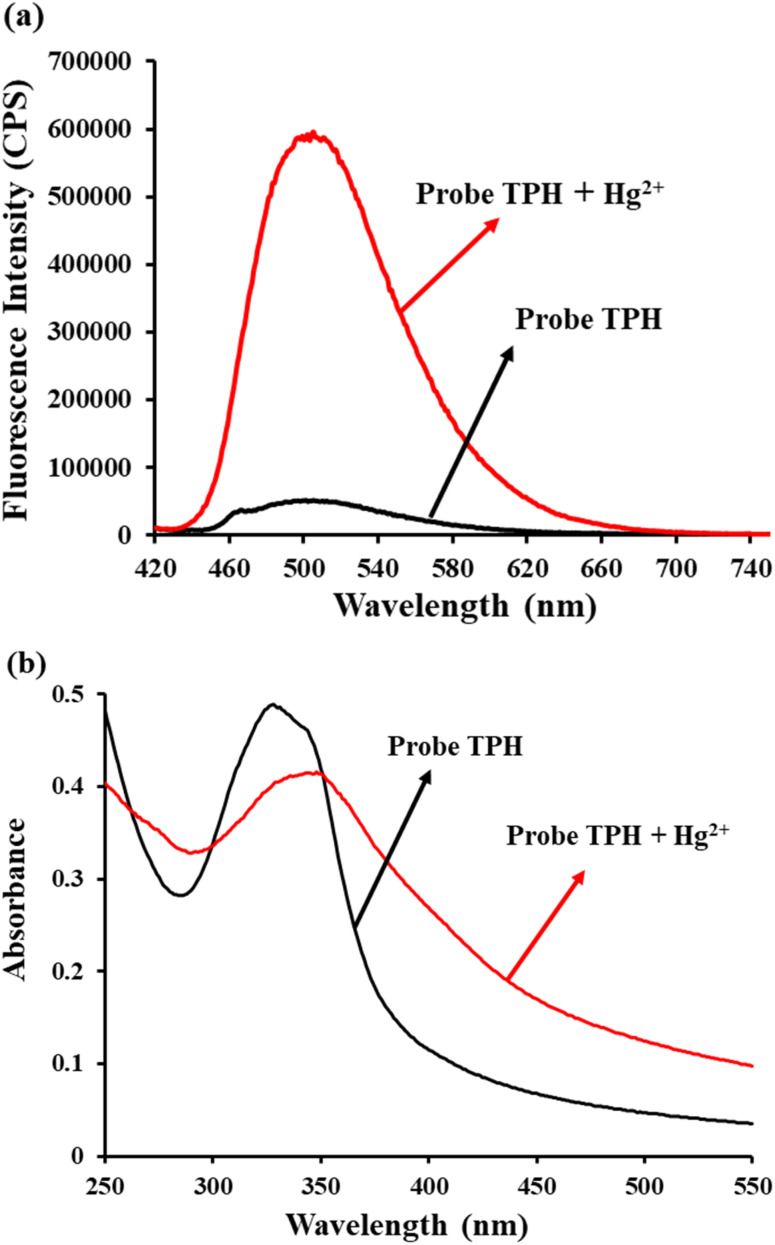
(a) Fluorescence and (b) absorption spectrum changes of TPH probe (5 μM and 20 μM, respectively) before and after the addition of Hg^2+^ (20 μM). *λ*_ex_ = 400 nm and *λ*_em_ = 505 nm. Conditions: HEPES (5 mM, pH 7.4).

**Scheme 2:**
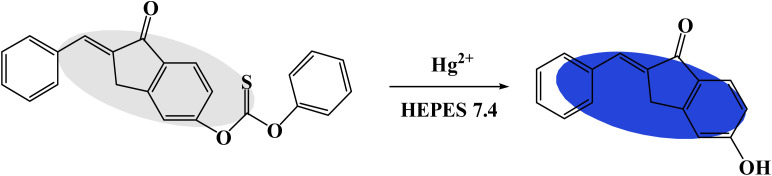
Recognition mechanism of TPH probe for Hg^2+^.

All the reactions were carried out in aqueous solution (HEPES 5 mM, pH = 7.4). The fluorescence spectra of the TPH probe for monitoring Hg^2+^ was investigated. When Hg^2+^ (20 μM) was added, the fluorescence intensity displayed a significant enhancement at 505 nm (Fig. 1a). The quantum yield of the TPH probe was calculated to be 0.07. Then, its absorption spectrum was also studied. According to the results, the absorption peak changed from 325 nm to 350 nm (Fig. 1b), implying that Hg^2+^ could promote the splitting of the carbonothioate moiety (Scheme 2). Furthermore, we conducted HRMS and NMR to explore the reaction mechanism of the TPH probe and Hg^2+^ (Fig. S1–S4 in the ESI†).

### 3.2 Quantification of Hg^2+^

**Fig. 2:**
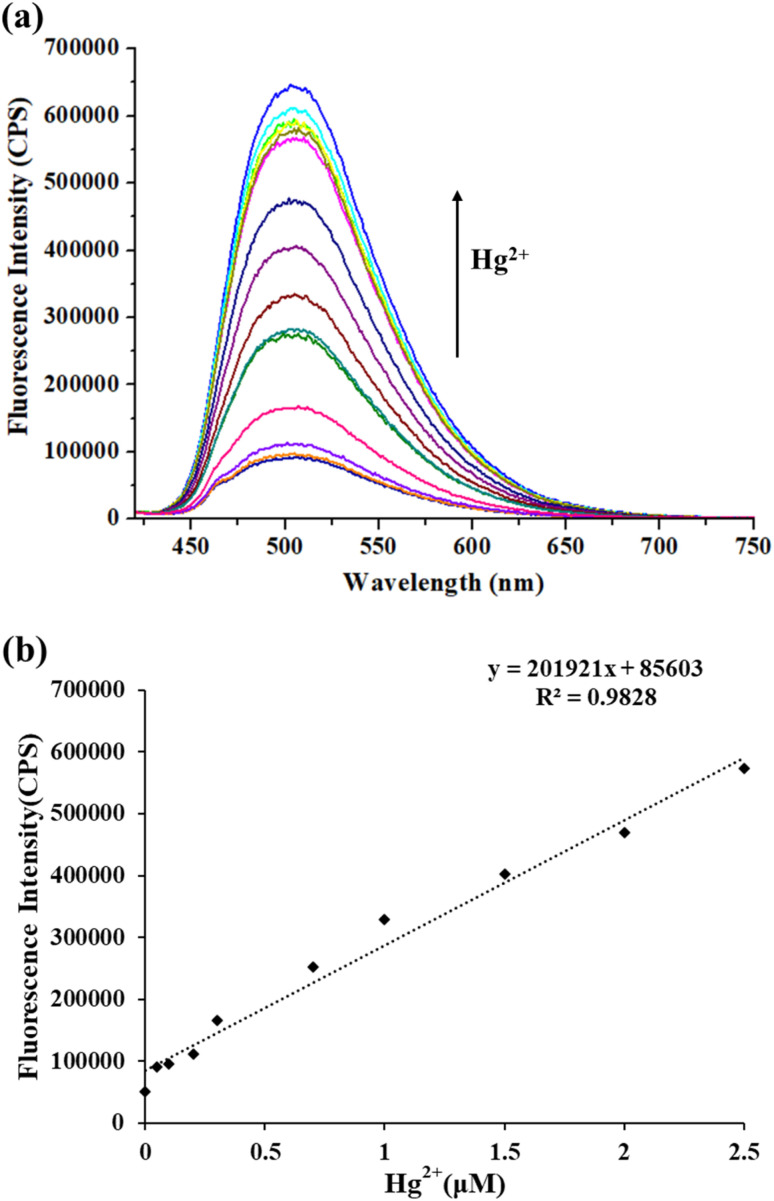
(a) Fluorescence spectra of TPH probe (5 μM) for Hg^2+^ (0–5 μM). (b) Linear plot of fluorescence intensity (505 nm) to Hg^2+^ (0–2.5 μM). *λ*_ex_ = 400 nm and *λ*_em_ = 505 nm. Conditions: in HEPES (5 mM, pH 7.4).

The TPH probe showed good water solubility, and thus the influence of the concentration of Hg^2+^ on its fluorescence intensity in pure water was investigated. With an increase in Hg^2+^ concentration (0–5 μM), the fluorescence intensity of the TPH probe at 505 nm increased accordingly (Fig. 2a). In addition, when the concentration of Hg^2+^ was 0–2.5 μM, it was linearly correlated with the fluorescence intensity (*y* = 201 921 [Hg^2+^] (μM) + 85 603, *R*^2^ = 0.9828) (Fig. 2b), and the LOD was 16 nM (3*σ*/*k*). Thus, all the above-mentioned results indicate that the TPH probe can provide a sensitive detection tool for Hg^2+^ in the actual water environment.

### 3.3 Specificity for Hg^2+^

**Fig. 3:**
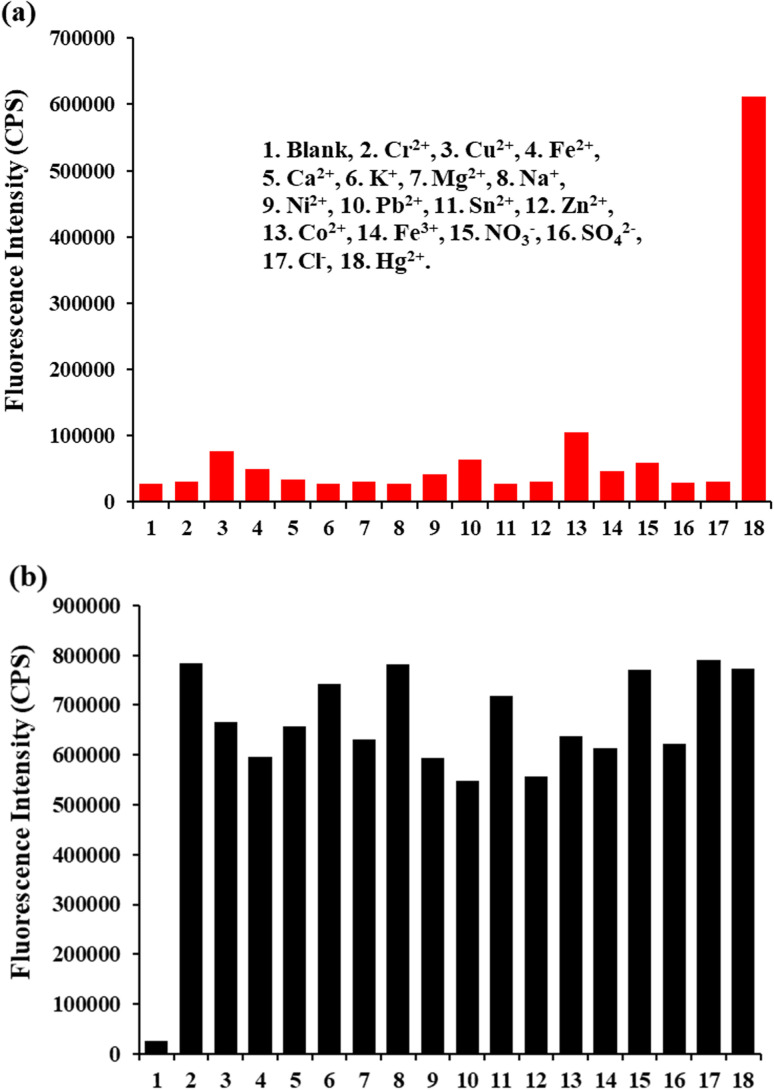
(a) Fluorescence response of TPH probe (5 μM) toward Hg^2+^ (5 μM) and other ions (50 μM). (b) Fluorescence response of TPH probe (5 μM) toward Hg^2+^ (5 μM) in the presence of other ions (50 μM). *λ*_ex_ = 400 nm and *λ*_em_ = 505 nm. Conditions: in HEPES (5 mM, pH 7.4).

The specificity of the TPH probe toward Hg^2+^ and other various relevant analytes including Cd^2+^, Cu^2+^, Fe^2+^, Ca^2+^, K^+^, Mg^2+^, Na^+^, Ni^2+^, Pb^2+^, Sn^2+^, Zn^2+^, Co^2+^, Fe^3+^, NO^3−^, SO_4_^2−^ and Cl^−^ was analyzed. The concentration of Hg^2+^ and the other analytes was 5 μM and 50 μM. Only Hg^2+^ caused a fluorescence response at 505 nm, while the other relevant analytes did not cause obvious fluorescence changes (Fig. 3a). Besides, interference experiments were also conducted. The fluorescence intensity response values exhibited a slight change at 505 nm (Fig. 3b). Thus, all these results strongly suggest that the TPH probe can specifically recognize Hg^2+^.

### 3.4 Analytical applications in real water samples

Then, the analytical application of the TPH probe in three water samples (lake water, underground water and river water) for the detection of Hg^2+^ was investigated. Firstly, no Hg^2+^ was found in the samples. Then, after 5 μM TPH probe was added to the test water samples, 1 and 2 μM Hg^2+^ were also respectively added. Each sample was tested three times. As can be seen in Table 1, the recoveries of the three water samples were 84.07–109.20%, further confirming that this newly synthesized probe could effectively detect Hg^2+^ in the real water environment.

**Table 1:** Application of TPH probe in three water samples^a^

Real water samples	Found Hg^2+^	Addition Hg^2+^ (μM)	Found (μM)	Recovery (%)	RSD (*n* = 3) (%)
Sample A	ND	1	0.97 ± 0.03	96.93	3.08
		2	2.01 ± 0.13	100.38	6.40
Sample B	ND	1	1.09 ± 0.06	109.20	6.42
		2	1.68 ± 0.13	84.07	6.64
Sample C	ND	1	0.96 ± 0.08	95.97	8.41
		2	1.72 ± 0.11	85.81	5.40

a ND: not detected. Sample A from JiaZi Lake, University of Jinan and samples B and C from Jinyun River and Jinyang River in Jinan, China.

## 4 Conclusions

The fluorescent TPH probe with phenyl thiochloroformate as the Hg^2+^ recognition site was synthesized in this study. This probe could specifically recognize Hg^2+^ and quantitatively detect Hg^2+^ in aqueous solution. According to the experimental results, we calculated that its detection limit is 16 nM. Meanwhile, the TPH probe has excellent water solubility, which is conducive for its application in the actual environment.

## Data availability

The datasets generated and analyzed during the current study are available from the corresponding author on reasonable request.

## Conflicts of interest

The authors have no conflicts of interest to declare.

## Supplementary Material

RA-013-D3RA02791K-s001
