# Supplementary material for: Highly selective fluorescent probe for detecting mercury ions in water
Source: RSC Adv. 2023 Jun 23;13(28):19091–5. doi: 10.1039/d3ra02791k (PMC10288340; doi:10.1039/d3ra02791k)
Supplement: RA-013-D3RA02791K-s001 [file RA-013-D3RA02791K-s001.pdf]

Electronic Supplementary Material (ESI) for RSC Advances.
This journal is © The Royal Society of Chemistry 2023

Supporting Information

Highly selective fluorescent probe for detecting mercury ion in water

Yanfeng Shi[a], Bingxu Li[a], Zhifeng Wang[a], Yanhao Zhang[a], Zhibin Zhang[a], Xu Zhang [b*], Fulin Li[c*]

[a] *School of Municipal and Environmental Engineering, Shandong Jianzhu University, Jinan, China*

[b] *School of Architecture and Urban Planning, Shandong Jianzhu University, Jinan, China*

[c] *Water Resources Research Institute of Shandong Province, Jinan, China*

*Corresponding author. * X. Zhang: Tel +86 156 6830 3582; E-mail: 15668303582@163.com*

** F. L. Li: E-mail: fulinli@126.com.*

Table of Contents

1. HRMS of probe **TPH** and reaction products of probe **TPH** and Hg^{2+}

2. ^{1}HNMR and ^{13}CNMR data of probe **TPH**

3. Effects of pH on the recognition of probe **TPH** for Hg^{2+}

4. The emission of the probe **TPH** at different excitation wavelengths

5. The stability of the probe **TPH** in water

6. The forms of salts were used of the metal ions

1. HRMS of probe TPH and reaction products of probe TPH and Hg^{2+}

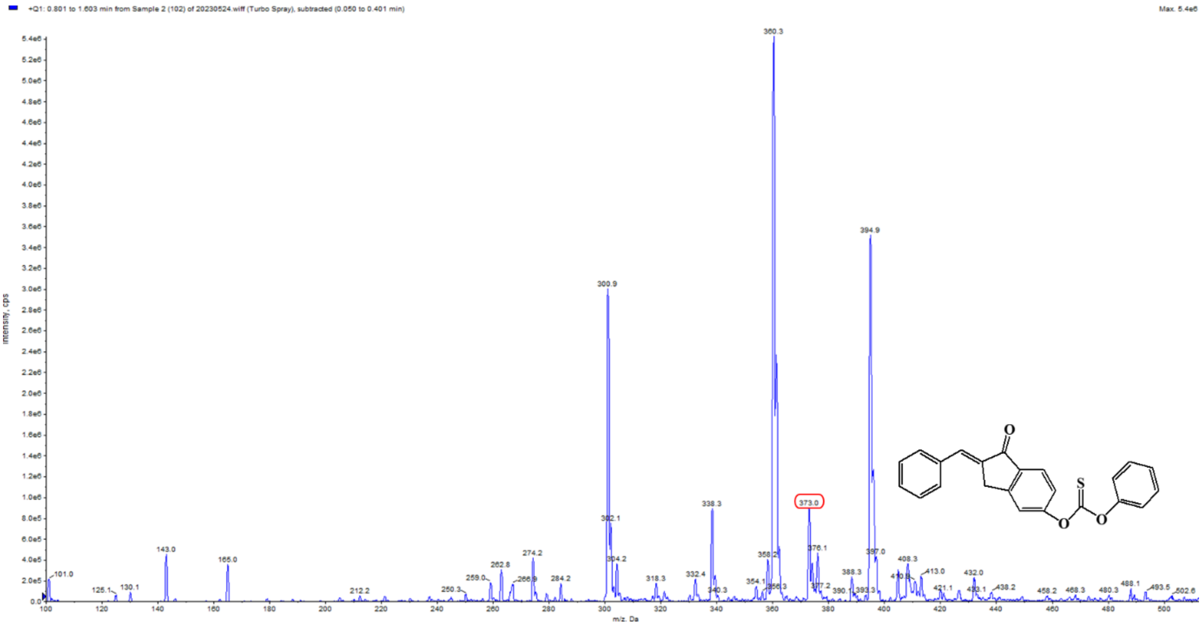

Figure S1. The HRMS data of probe **TPH**

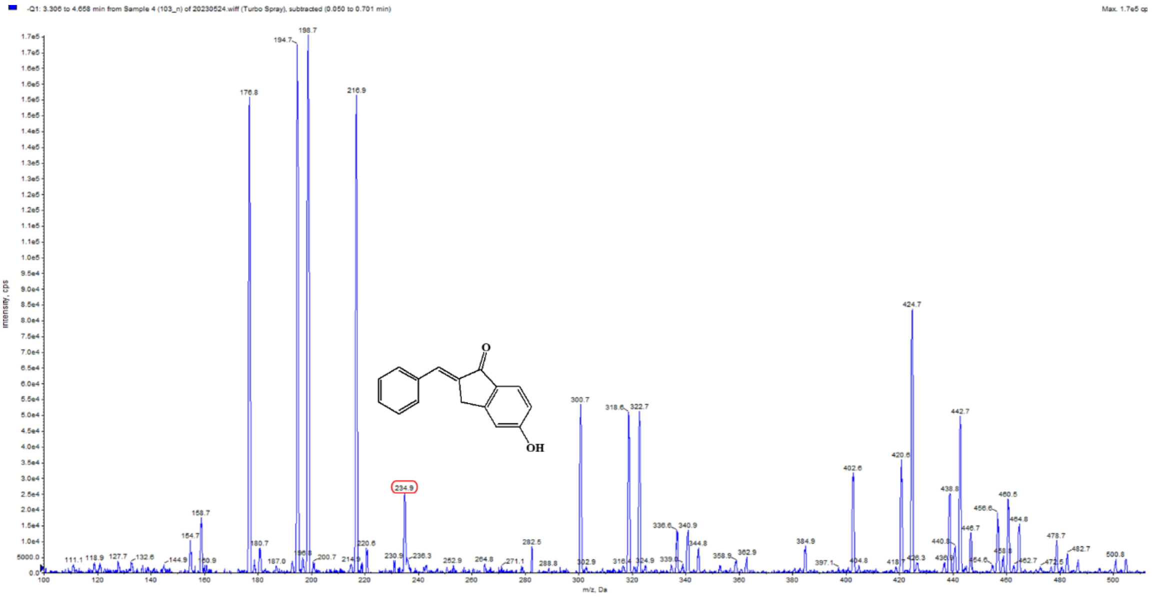

Figure S2. The HRMS data of the reaction products of probe **TPH** and Hg^{2+}.

2. ^{1}HNMR and ^{13}CNMR data of probe TPH

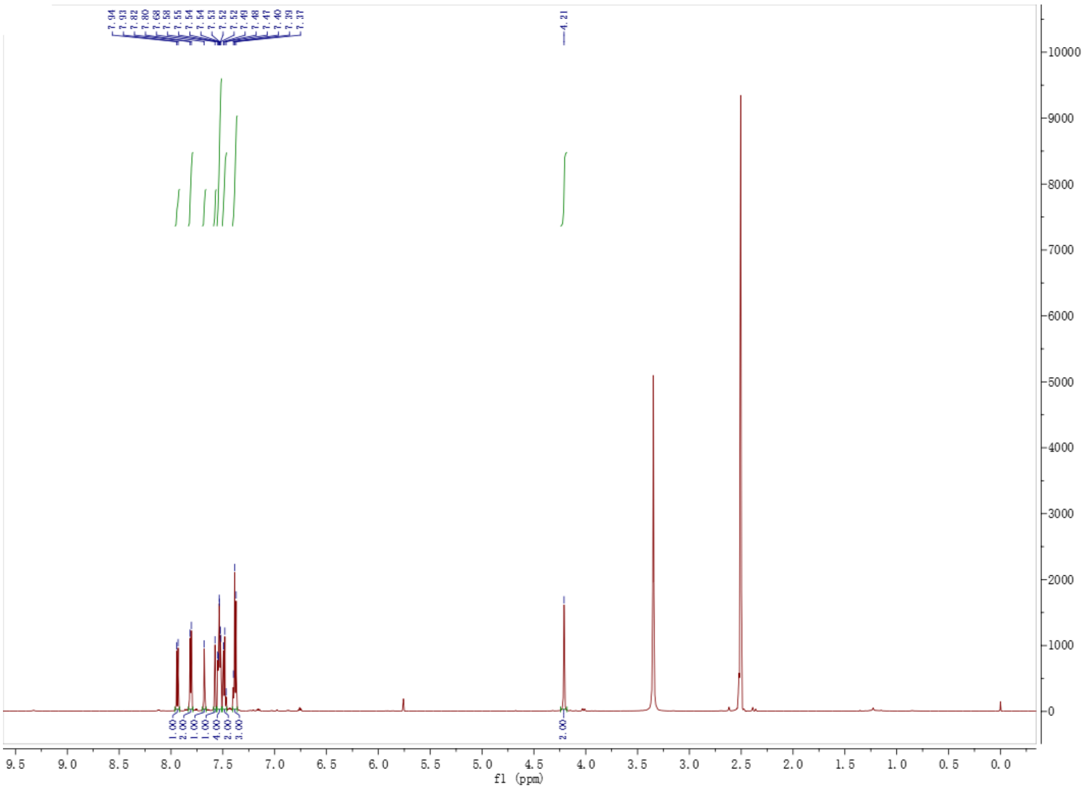

Figure S3. ^{1}H-NMR data of probe **TPH**.

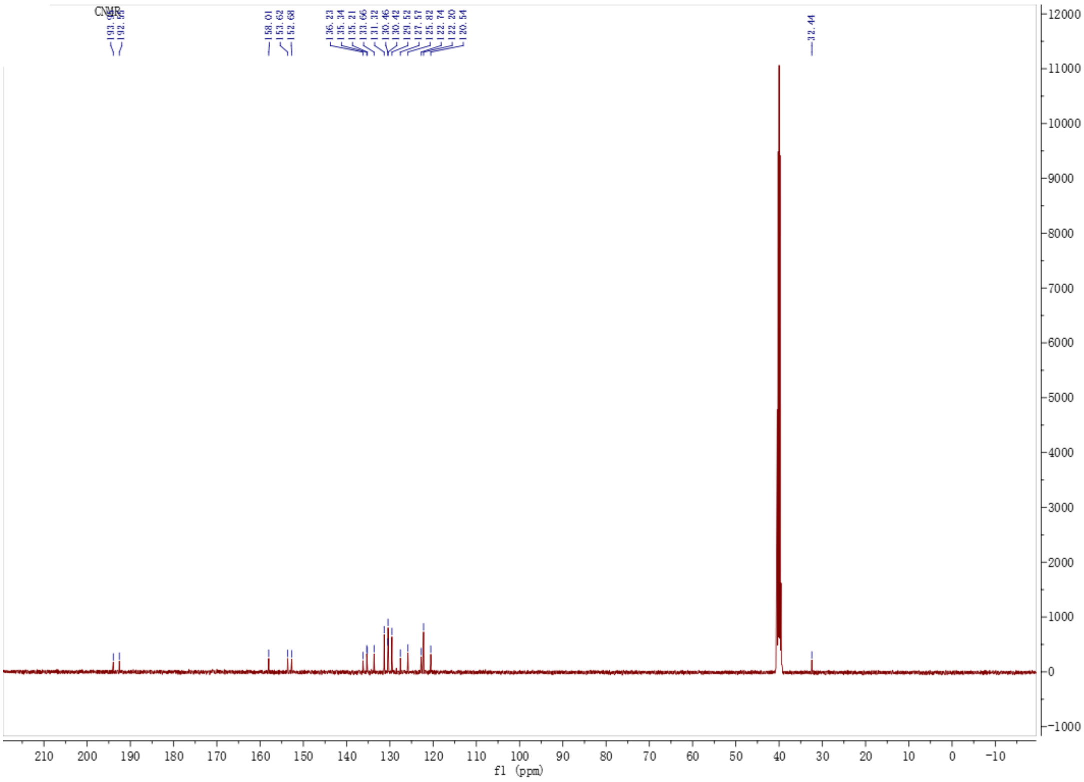

Figure S4. ^{13}CNMR data of probe **TPH**.

3. Effects of pH on the recognition of probe TPH for Hg^{2+}

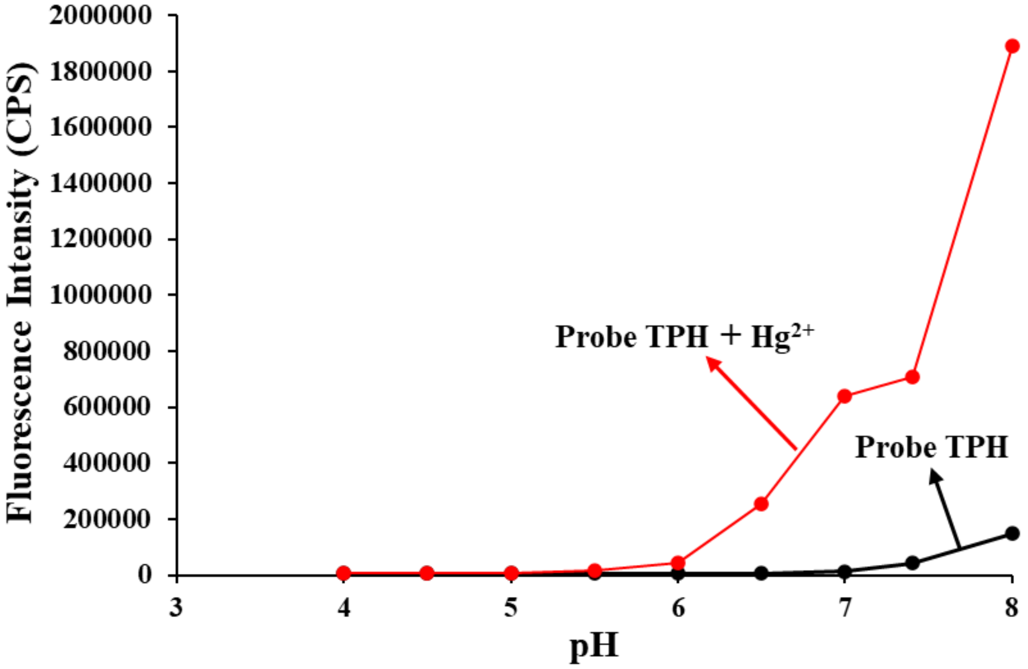

Figure S5. The effects of pH on probe **TPH** (5 μM) for detecting Hg^{2+} (20 μM).

4. The emission of the probe TPH at different excitation wavelengths

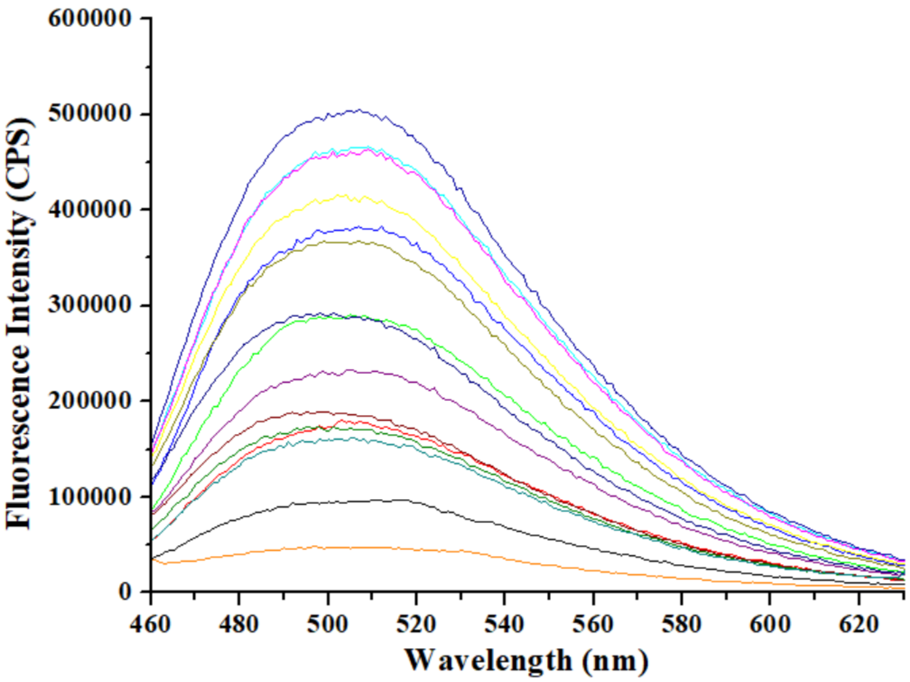

Figure S6. The emission of the probe TPH at different excitation wavelengths (320-450 nm)

5. The stability of the probe TPH in water

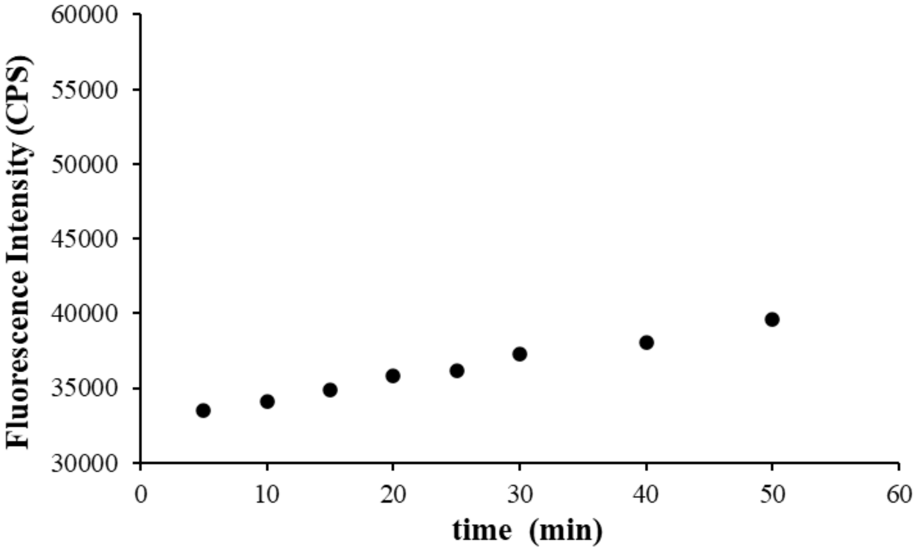

Figure S7. The stability of the probe **TPH** in water

6. The forms of salts were used of the metal ions.

The forms of salts were used in this paper: $MgSO_4$, $CoSO_4$, $NiSO_4$, $MnSO_4$, $ZnCl_2$, $CuSO_4$, NaCl, $AgNO_3$, $CaCl_2$, $Pb(NO_3)_2$, $CdSO_4$, $AlCl_3$, $FeSO_4$, $Fe_2(SO4)_3$, KCl, $SnCl_2$, $CrSO_4$.
